# Methods of identifying surgical Necrotizing Enterocolitis—a systematic review and meta-analysis

**DOI:** 10.1038/s41390-024-03292-3

**Published:** 2024-06-07

**Authors:** George S. Bethell, Ian H. Jones, Cheryl Battersby, Marian Knight, Nigel J. Hall

**Affiliations:** 1 University Surgical Unit, Faculty of Medicine, University of Southampton, Southampton, UK; 2 Department of Paediatric Surgery and Urology, Southampton Children’s Hospital, Southampton, UK; 3 Department of Paediatric Surgery and Urology, Birmingham Children’s Hospital, Birmingham, UK; 4 Neonatal Medicine, School of Public Health, Faculty of Medicine, Imperial College London, London, UK; 5 Nuffield Department of Population Health, National Perinatal Epidemiology Unit, University of Oxford, Oxford, UK

## Abstract

**Background:**

Current data suggests potential benefit of earlier surgery for necrotizing enterocolitis (NEC) however this requires accurate prognostication early in the disease course. This study aims to identify and determine the effectiveness of previously reported methods or tests for the identification of surgical NEC.

**Methods:**

Systematic review and meta-analysis with registration on PROSPERO including articles describing a method of identifying surgical NEC. Outcomes of interest were effectiveness and repeatability of index test.

**Results:**

Of the 190 full-text articles screened, 90 studies were included which contained 114 methods of identifying surgical NEC in 9546 infants. Of these methods, 44 were a scoring system, 37 a single biomarker, 24 an imaging method, and 9 an invasive method. Sensitivity and specificity ranged from 12.8–100% to 13–100%, respectively. Some methods (9.6%) provided insufficient methods for repeatability within clinical practice or research. Meta-analyses were possible for only 2 methods, the metabolic derangement 7 score and abdominal ultrasound.

**Conclusions:**

A range of methods for identifying surgical NEC have been identified with varying overall performance and uncertainties about reproducibility and superiority of any method. External validation in large multicentre datasets should allow direct comparison of accuracy and prospective study should evaluate impact on clinical outcomes.

**Impact:**

- Earlier identification of need for surgery in necrotizing enterocolitis (NEC) has the potential to improve the unfavourable outcomes in this condition. As such, many methods have been developed and reported to allow earlier identification of surgical NEC.
- This study is the first synthesis of the literature which identifies previously reported methods and the effectiveness of these.
- Many methods, including scoring systems and biomarkers, appear effective for prognostication in NEC and external validation is now required in multicentre datasets prior to clinical utility.

## Introduction

A quarter of babies with necrotizing enterocolitis (NEC) will require surgery due to bowel perforation, suspected bowel necrosis or failure to improve from medical treatment alone.^1^ In those that do undergo laparotomy, the most common finding is ischaemic, non-viable gut which requires resection.^2^ In 1-in-20 surgical procedures, the extent of the non-viable gut means that survival is not possible.^2^ In addition, population based data demonstrate that 20% of infants with NEC die without having a surgical procedure.^3^ It is possible that some of these deaths might be avoidable and findings at surgery may be less advanced with earlier recognition of advanced disease. Hence earlier escalation of treatment, including surgery, has potential to interrupt the inflammatory cascade through resection of necrotic bowel. Given that existing data suggest that treatment delay is associated with worse outcomes,^4^ we hypothesise that earlier identification of need for treatment escalation, including surgery, in infants with NEC is likely to improve the currently unfavourable outcomes.

A recent analysis of a population based dataset of infants with surgical NEC explored the relationship between indication for surgery, timing of surgery, and outcomes.^4^ This revealed that babies who have subjectively been felt to have failed medical management have surgery 30 h later than babies who have a more definite indication for surgery (i.e., the presence of pneumoperitoneum). This delay to surgical intervention was associated with 4.5 times greater risk of poor outcome (death or requirement for parental nutrition at 28 days following surgery).^4^ This group of babies represents a third of those who do undergo surgery and are of particular interest, since if we can identify the need for surgery earlier this may pave the way to improving outcomes.^4^ To enable this, a mechanism for early identification of infants with surgical NEC would be needed. Such a mechanism should be objective, accurate, and easily implementable to overcome the barriers presented by current decision-making in these infants. A successful method or predictive tool would identify babies soon after they develop critical intestinal ischaemia or necrosis and produce a result rapidly enough to allow escalation of treatment that likely includes early surgery. If available, such a mechanism may predict which infants would benefit from surgical intervention and facilitate an earlier operation, which has the potential to positively impact outcomes.

Various scoring systems, biochemical biomarkers, imaging methods, and invasive techniques have been reported to identify infants with surgical NEC with differing degrees of effectiveness.^5–8^ One example of a method that has been proposed as useful is the metabolic derangement 7 (MD7) score which combines vital signs with laboratory data to predict the need for surgery.^5^ Yet neither this nor other techniques are in routine use in a large number of centres. Overall, it is not known how many methods exist, nor which are most effective. Applicability of any developed method to clinical practice is also important to consider and is an essential consideration when implementing into clinical practice.^9^

The aim of this study was to identify which methods or tests have been investigated to identify surgical NEC, which are most effective, and additionally determine which methods have or can be implemented into clinical practice, based on adequate description of the method.

## Methods

### Study design

A systematic review was conducted as per recommendations by the preferred reporting items for systematic reviews and meta-analysis (PRISMA) guidance.^10^ The full methodology was registered as a protocol on PROSPERO (CRD42022365090) prior to commencing the literature search, on 7th October 2022.

### Literature search

Medline, Embase, Scopus, Web of Science, and the Cochrane library were searched on 21st October 2022 for terms “Necrotising Enterocolitis” AND “surgical” OR “surgery” AND “biomarker” OR “identification” OR “test” OR “diagnosis” OR “severity”. MeSH terms were used to ensure all variations of these terms were used. The full search strategy is included in the supplementary material. All languages were sought however conference abstracts not leading to peer reviewed publication and case reports were excluded. Grey literature and references of included studies were all scrutinised for additional studies. Duplicate studies were excluded.

### Study selection

Abstracts were independently screened by two reviewers with conflicts resolved by discussion. Full texts were then sought for abstracts deemed worthy of full text review. Studies were included if they reported a method or test which discriminates babies with surgical NEC, from those with medical NEC, or reported a method or test which identifies severity of NEC, with at least one measure of test effectiveness (e.g., sensitivity, specificity, positive predictive value, negative predictive value). Surgical NEC was defined as infants with NEC who had undergone surgical intervention. When infants who died of NEC were included in the surgical NEC group in the source study this was noted. Studies were excluded if their focus was on infants with spontaneous intestinal perforation or compared surgical NEC only to those without NEC (healthy controls). Studies that reported only an association between a feature and surgical NEC, without providing a measure of test or method effectiveness, were also excluded. Additionally, those that described a combination of features used in clinical practice, without testing the effectiveness of this approach were excluded. This latter point mainly relates to more historical literature.

All studies that met these criteria were included and further categorised based on the type of method or methods investigated. Studies that investigated multiple methods were included in the same category more than once or in multiple categories as appropriate. Methods which combined more than one feature into a scoring system were categorised as a ‘scoring system’. Single biomarker methods were those using a single biomarker, either molecular, histological, or physiological. Imaging methods included methods which solely used imaging without addition of any other clinical data or biomarker. Invasive methods included techniques such as paracentesis and laparoscopy, again without clinical data or additional biomarkers.

### Data extraction

Data were extracted from included studies, and categorised, using a standardised data collection tool and stored within Review Manager version 5.4 (Cochrane, Nordic Cochrane Centre, Copenhagen, Denmark). Studies were translated into English, when required, prior to this. Data extracted included study details, study design, reference test definition and timing, index test definition and timing, effectiveness of method, sample size, and inclusion/exclusion criteria. The population of infants included in the study were taken as a whole without separation of sub-groups such as presence of pneumoperitoneum, or not.

### Outcomes

The main outcomes of interest were test effectiveness summarised as sensitivity, specificity, positive predictive value (PPV), negative predictive value (NPV), and area under receiver operating characteristic curve (AUC). These were taken for each time point provided if a test was repeated. If a study did not provide these outcomes but did provide sufficient results to calculate these (true positives, false positives, true negatives, false negatives), then these outcomes were calculated.

### Bias and applicability assessment

Bias and applicability of included methods was assessed using the Quality Assessment of Diagnostic Accuracy Studies (QUADAS-2) tool.^11^ Risk of bias was determined using specified considerations within the QUADAS-2 tool which focussed on aspects of study design, results, and interpretation. This was based on four domains which were patient selection, index test, reference standard, and flow/timing. Applicability concerns were also assessed using specified considerations within QUADAS-2 and included three domains; patient selection, index test, and reference standard. This part of the assessment focussed on the applicability of the reported method to future research and potential clinical use, which includes provision of sufficient, reproducible methods regarding the index test. Risk of bias and applicability plots were then created for each category of method and then all included studies.

### Data synthesis

Effectiveness of included methods were summarised in tables with repeated methods presented together. Where multiple studies reported the same test, meta-analysis was undertaken to provide pooled sensitivity and specificity for these methods using random effects models. As per the Cochrane handbook for diagnostic test accuracy study, meta-analysis was not conducted where different thresholds were used for the same test or if a study reported insufficient data to allow this. Where a study had not assessed test effectiveness but instead provided association of imaging features with disease severity, 2 × 2 tables were synthesised using random effect modelling and reported as odd ratios with 95% confidence intervals (CI) using Review Manager version 5.4 (Cochrane, Nordic Cochrane Centre, Copenhagen, Denmark).

## Results

### Search results

**Fig. 1:**
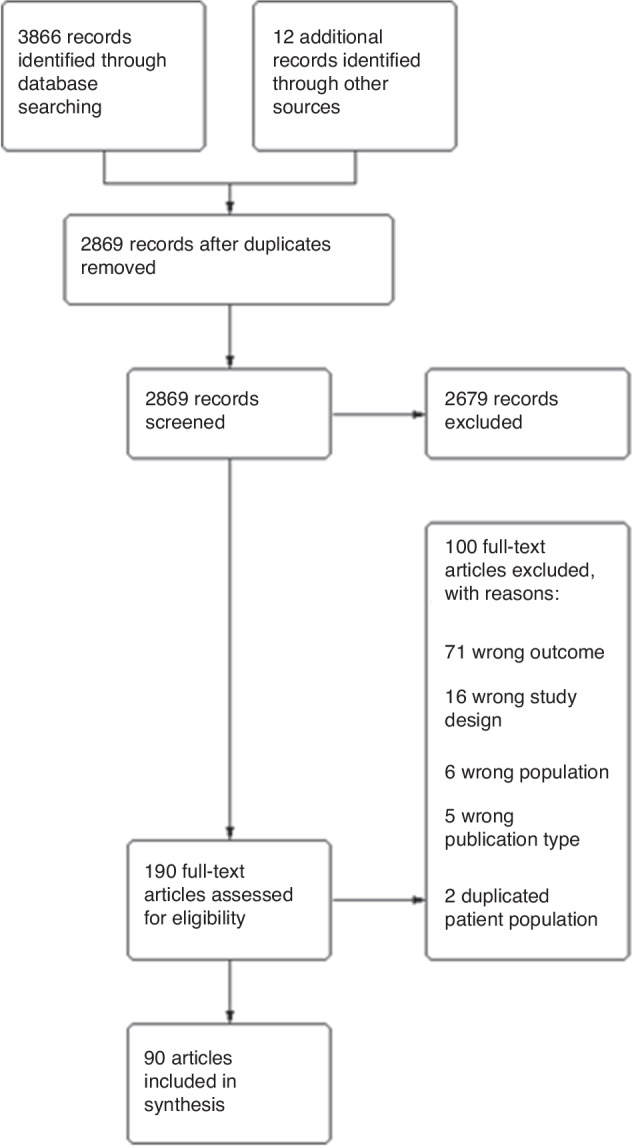
PRISMA flow diagram. Diagram detailing literature search, article screening and full-text assessment.

There were 3866 articles identified in the literature search and 12 via reference checking of which there were 2869 after removal of duplicates (Fig. 1). From these 190 underwent full text assessment by two reviewers.

### Included studies

The were 90 articles included which contained 114 methods of identifying surgical NEC with data derived from a total of 9546 infants. Of these 3552 (37.2%) infants had surgical NEC. The majority of the methods (81.6% [93/114]) were derived or tested in single centre articles and 60.5% (69/114) were produced and tested retrospectively. In regards to inclusion criteria the most commonly (64.9% [74/114]) used definition was Bell’s stage II and III or equivalent, which was definite radiological evidence of NEC (i.e., pneumatosis or portal venous gas). Only 19.2% (22/114) of methods reported specific exclusion of infants with spontaneous intestinal perforation (SIP). Exclusion of infants with pneumoperitoneum occurred in 17.5% (20/114) of  methods with the aim of producing a method applicable to infants with NEC but without pneumoperitoneum since pneumoperitoneum is a universally accepted absolute indication for surgery.^12^

**Fig. 2:**
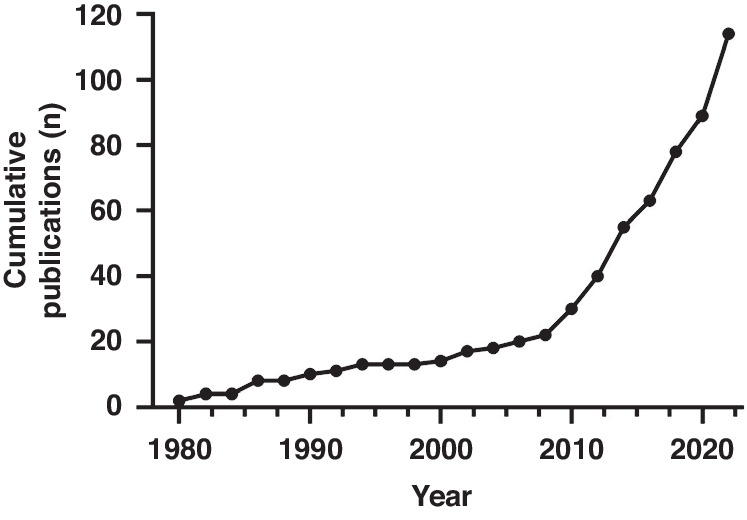
Cumulative publications per year of included studies (1979–2022). This shows a significant increase in number of publications during recent years.

Only 8.7% (10/114) methods used separate training and testing cohorts of infants to develop and validate a method. Most reports provided sufficient detail to repeat the method however this was not possible for 9.6% (11/114) of methods. The number of reported publications has increased significantly over time particularly from years 2010 to 2022 (Fig. 2).

### Methods identified

Of the methods, there were 44 that were categorised as a clinical scoring system, 37 as a single biomarker, 24 as an imaging method, and 9 as an invasive method.

Identical or similar methods were reported by multiple articles and a summary of all methods is shown in Table 1. Ultrasound^13–23^, the metabolic derangement 7 score (MD7)^5,24–29^, Intestinal fatty acid-binding protein^8,30–33^, plasma C-reactive protein (CRP)^34–38^ and laparoscopy^7,39–42^ were all evaluated in at least 5 studies. The majority (34/44) of methods using a clinical scoring system did not appear in multiple studies whereas only 8/37 single biomarker studies were not repeated.

**Table 1:** Summary of included studies which identify surgical versus medical necrotizing enterocolitis.

*Index test*	*Years, range*	*Medical, n*^*$*^	*Surgical, n*^*$*^	*Published methods, n*	*Article references*
*Clinical scoring systems*					
*MD7 score*	2010–2022	395	227	7	5,24–29
*SNAPPE-II score*	2012–2022	150	75	3	25,29,43
*Other scoring systems*	1979–2022	2829	1603	34	26–28,43–56,85–99
*Single biomarker methods*					
*Intestinal fatty acid-binding protein*	2010–2020	83	80	7	8,30–33
*Plasma CRP*	2010–2022	294	108	5	34–38
*Interleukin 6*	1994–2020	41	37	4	34,36,57,58
*Platelets*	2001–2022	50	159	3	59,60
*Serum amyloid A*	2011–2020	23	19	2	36,61
*Procalcitonin*	2018–2022	267	59	2	37,38
*Heart rate*	2013–2018	77	65	2	62,63
*Lactate*	2010–2022	149	72	2	35,38
*Red cell T-Cryptantigen activation*	1986–2002	72	94	2	64,65
*Other single biomarkers*	2011–2022	366	253	8	34,36,38,100–103
*Imaging methods*					
*Ultrasound*	1986–2018	414	248	11	13–23
*Abdominal radiograph*	1982–2020	175	123	4	15,69–71
*Ultrasound and AXR*	2011–2016	250	87	3	66–68
*DAAS AXR score*	2009–2022	275	99	3	28,72,73
*Other imaging methods*	1981–2021	38	27	3	74–76
*Imaging methods*					
*Laparoscopy*	2004–2011	7	36	5	7,39–42
*Paracentesis*	1980–1990	30	67	3	77–79
*Intravesical pressure*	2013	9	14	1	80
*TOTAL*	1979–2022	5994	3552	114 methods	90 articles

*MD7* metabolic derangement 7, *DAAS* Duke abdominal assessment scale, *SNAPPE-II* Score for Neonatal Acute Physiology Perinatal Extension-II, *$* some children in multiple rows as some articles report multiple index tests, *n* number of infants.

### Bias and applicability

**Fig. 3:**
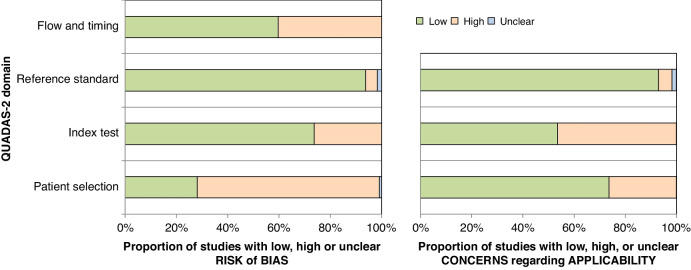
Bias and applicability summary of included studies. Risk of bias assessment for each of the four domains and applicability concerns for each of the three domains are shown as a proportion of all included articles. Green, red and blue areas represent low, high and unclear risk/concern respectively.

Bias and applicability of included methods were variable (Fig. 3). High risk of bias was seen in the patient selection domain for many methods due to unbalanced group size and retrospective design. This was greatest in studies reporting single biomarker methods. Risk of bias regarding the index test was generally low however higher in clinical scoring system and single biomarker studies due to many studies providing insufficient methodology. Risk of bias in the reference standard domain was generally low as surgical NEC (and/or death) were clearly defined. Bias risk regarding patient flow and timing was assessed as high for many studies due to inconsistency in timing regarding the application of a test or components of it.

Applicability concerns regarding patient selection arose in a similar proportion of studies from all four categories and was mainly associated with inclusion of infants without definite NEC, i.e., Bell’s stage I. High applicability concerns related to the index test generally arose due to provision of results from a new prediction method without sufficient methods for the prediction method to be validated externally or used clinically. This was most common in studies reporting machine learning methods to derive scoring systems. Applicability concerns regarding the reference standard were low due to a clearly defined population of infants as per the inclusion criteria set out in the search strategy.

### Clinical scoring systems

There were 44 reports identified of 36 unique clinical scoring systems (Supplementary Table 1).

#### Metabolic derangement 7 (MD7) score

There were seven reports of development or evaluation of the MD7 score.^5,24–29^ This score was first described in 2010^5^ and consists of 7 criteria which score 1 point each. This score is designed for infants who do not have pneumoperitoneum. It is not clear exactly how this score was developed and a score of 3 or more is stated to warrant surgical intervention, unless there is a good indication determined by the clinician that this is not required. The first two reports of the MD7 score focussed on overall clinical outcome, rather than the ability of the test to identify surgical NEC. These first two studies reported that a score of 3 or more was associated with a poor overall clinical outcome and also that the use of MD7 in a neonatal unit improves outcomes compared to a unit where it was not used (supplementary Table 2).^5,24^ Further reports^25–29^ focussed on test effectiveness at identifying surgical NEC, or surgical NEC and death, with AUC as high as 0.77 and sensitivity and specificity as high as 92% and 83.1% respectively (Supplementary Table 2) using a cut-off score of 3. One study changed the threshold of the test as they found that a score of 2 provided the highest AUC.^29^ Two studies^27,28^ combined the MD7 score with another score in an attempt to provide a more effective method. Khalak et al.^27^, in a multicentre prospective study, found that the MD7 score combined with an additional 7 feature clinical score was more discriminatory than the MD7 score alone with an AUC of 0.89 compared with 0.74. Yu et al.^28^ reported that the MD7 score combined with the Duke Abdominal Assessment Scale (DAAS) provided a specificity and PPV of 100% however a sensitivity of only 12.8%. The authors concluded that this method was poor and that ultrasound alone was much more effective at identifying patients requiring surgery. Pooling of test effectiveness using meta-analysis was only possible for two studies reporting the MD7 score, both of which were single centre and retrospective, due to provision of incomplete measures of test effectiveness or use of a different positive test threshold^26,28^. It was found that the estimated sensitivity and specificity from this was 0.77 (95% CI 0.38 to 0.95) and 0.73 (95% CI 0.47 to 0.89) respectively using a threshold of ≥3.

#### Score for neonatal acute physiology perinatal extension-II

The Score for Neonatal Acute Physiology Perinatal Extension-II (SNAPPE-II) was reported in 3 retrospective, single centre studies.^25,29,43^ Each study found that a different threshold was most effective at identifying surgical NEC (Supplementary Table 1). Results were similar between studies, with AUC reported between 0.69-0.71 (Supplementary Table 2). One of these studies^29^ found that SNAPPE-II was not as effective as the MD7 score however concluded that both scoring systems are useful in NEC. Ibanez et al.^25^ also compared SNAPPE-II to the MD7 score and found no statistically significant difference between either test at two time points but concluded that neither test should be used to guide decision making in NEC due to unfavourable test effectiveness (Supplementary Table 2). Lin et al.^43^ compared the SNAPPE-II to Score for Neonatal Acute Physiology-II (SNAP-II) and found that SNAP-II was marginally more effective than SNAPPE-II (AUC = 0.75 vs 0.71) but concluded that both methods should be used in clinical practice.

#### Other scoring systems

The other methods using clinical scoring systems were not repeated in more than one study. One study^44^ that implemented machine learning techniques to develop a method used 49 clinical and radiological features from a single centre, retrospective dataset. This method was reported as highly effective with an AUC of 0.94 and similarly high other measures of effectiveness (Supplementary Table 2) whilst it was shown to be superior to senior clinicians at decision making. Despite this, a clinically translatable description of the method was not provided. Insufficient methods to repeat the reported test was seen in several other studies^45–47^, most of which utilised machine learning for method derivation.

Another included study^48^ that was prospective and multicentre, compared two novel methods to produce both a clinical score and a clinical score combined with novel urinary peptides with validation in training and testing cohorts. This study included a large number of infants in the clinical method development but was limited by the number of urine samples available for analysis for the scoring system that included urinary peptides (Supplementary Table 1). Both methods were effective with an AUC of 0.89 in a training cohort and the combined method^48^, including the urinary peptides, correctly classified 100% of cases as either medical or surgical NEC. The final models were derived by machine learning and the provided test features are insufficient to reproduce these tests without access to the exact algorithm.

Rao et al.^49^ also developed a clinical scoring system using many readily available clinical features. It proved highly effective with sensitivity and specificity of 97.2% and 91.4% respectively (Supplementary Table 2). The reported advantages of this method is that it uses conventional statistics so the method can be tested in other cohorts easily or adopted clinically. The study was limited due to its single centre retrospective nature and it did not validate the method on a testing cohort of infants.

Kang et al. reported a further scoring system using readily available features which provides sufficient methods to be used clinically and was developed on a multicentre cohort of almost 150 infants with NEC.^50^ This method appears highly effective with a reported sensitivity and specificity above 90% and an AUC of 0.98. Further validation within a testing cohort was not described which is a limitation of this work. Lazow et al.^51^ reported a simpler method using only 4 features to identify infants with surgical NEC acutely or those that subsequently developed a NEC related stricture. This score relies on clinical examination, AXR, and ultrasound to produce a score from 0 to 4. Not all measures of test effectiveness are provided however this method achieved a sensitivity of 94.9% and 99.7% using a threshold of 3 and 4 points respectively (Supplementary Table 2). The authors conclude that this method has potential for clinical use in NEC without perforation. Some methods^52–56^ used only two features to produce a clinical prediction score. One of these^53^, prospectively used serum amyloid A concentration and platelet count and showed that it was possible to produce a prediction model with a sensitivity and specificity of 94% and 83% respectively, however only 29 infants were included. The authors concluded that prospective evaluation on larger numbers is required despite their promising results.

### Single biomarker methods

There were 37 reports identified, of 17 unique single biomarkers (Supplementary Table 3).

#### Intestinal fatty acid–binding protein

There were seven reports^8,30–33^ of the evaluation of urinary or plasma intestinal fatty acid–binding protein (i-FABP). All studies used a different threshold to define a positive test and two^31,32^ did not report what this was. Inclusion criteria were similar between studies and number of included infants ranged from 14 to 35. All these studies were prospective and the effectiveness of this method was generally good with AUC as high as 0.96 with a specificity of 100 reported by one study^8^ (Supplementary Table 4). Heida et al. was the only multicentre i-FABP study and also reported a positive linear correlation between length of resected bowel and both urinary and plasma i-FABP.^32^ Two studies^30,33^ compared effectiveness of urinary i-FABP to plasma i-FABP and came to different conclusions. El-Abd Ahmed et al.^30^ found that urinary i-FABP was more effective at identifying infants with surgical NEC than plasma i-FABP however Schurink et al.^33^ found the opposite. Each of the studies acknowledged that the clinical use of i-FABP in NEC is not currently practical as the analysis takes several hours and is not routinely available in healthcare pathology labs.

#### C-reactive protein

Plasma C-reactive protein (CRP) was investigated in five studies^34–38^ as a standalone biomarker. Study size was variable but above 100 infants in two studies^37,38^ (*n* = 142 and 184) and all used CRP at diagnosis except for one study^35^ which explored change in CRP from diagnosis to 72 h post diagnosis. The usefulness of CRP to detect surgical NEC varied between studies, a change of ≥390% from diagnosis to 72 h following this provided an AUC of 0.933^35^ however the AUC was as low as 0.25 in one study^34^ (Supplementary Table 4). Overall reports concluded that CRP is useful however none of the studies validated their findings in a separate cohort of infants.

#### Interleukin 6

Four studies^34,36,57,58^ investigated plasma or serum interleukin 6 of which the majority where prospective (Supplementary Table 3). These were smaller studies than most and where reported, each used different thresholds for a positive result. Reported AUC varied from 0.657 to 0.931 (Supplementary Table 4). It was also found that when repeated on days 3 and 7 after diagnosis the test was more effective at identifying those with surgical NEC.^34,36^

#### Platelet count

Platelet count was also investigated in two single centre, retrospective reports^59,60^. One study^60^ explored both rapid platelet fall (defined as >150 × 10^9^/L drop in 24 h reaching ≤100 × 10^9^/L) and severe thrombocytopenia (defined as 2 consecutive results <100 × 10^9^/L). It was found that the latter was moderately effective however rapid platelet fall provided the highest positive predictive value at 92% (Supplementary Table 4). The authors concluded that this method is useful in NEC however cannot be used as an isolated method to predict extent of disease. An additional study^59^ came to the same conclusion.

#### Serum amyloid A

Two relatively small prospective studies^36,61^ used serum amyloid A as a sole biomarker with similar reported measures of effectiveness. However, this method proved less effective at subsequent time points from diagnosis where investigated.^36^

#### Procalcitonin

Plasma procalcitonin use was also reported by two retrospective, single centre studies^37,38^, one of which contained over 180 infants. The threshold for a positive test differed between studies and reported effectiveness varied (Supplementary Table 4). Wang et al.^37^ compared use of procalcitonin to CRP and found CRP to be more effective at multiple time points. Another, much larger, study^38^ however found that procalcitonin was more effective than CRP, lactate, and fibrinogen at predicting surgical NEC at time of diagnosis.

#### Heart rate

Heart rate measures were investigated in two methods in the form of heart rate variability^62^ and mean heart rate^63^. Both methods were shown to be effective at identifying infants with NEC who require surgery to the extent that the heart rate characteristic index, a reflection of heart rate variability, increased up to 16 h prior to a diagnosis of NEC being made in the group that went on to have surgery. The heart rate characteristic index also increased 6 h prior to the diagnosis of NEC in the medical NEC group however the index was significantly higher in those with surgical NEC. The authors concluded that the particular advantage of this method is that it collects data continuously however further work is required to externally validate these methods.

#### Lactate

Lactate was investigated by two retrospective, single centre studies^35,38^ and Yu et al.^38^ found that it was not as effective as other single biomarkers already discussed in this section. Srinivasjois et al.^35^ investigated change in lactate and CRP from diagnosis to 48 or 72 h following this and also found that CRP was a more effective biomarker than lactate for this purpose. The authors conclude that both methods may be useful in this population.

#### Red cell T-Cryptantigen

Two studies^64,65^ investigated the significance of red cell T-Cryptantigen activation and found that this occurred more frequently in advanced disease. Unfortunately, provided test characteristics were insufficient to make a direct comparison between studies. Other methods that provided varying levels of reported effectiveness are shown in Supplementary Tables 3 and  4.

### Imaging methods

Seven different methods for identifying the need for surgery were identified in 24 studies (Supplementary Table 5).

#### Abdominal ultrasound

Eleven single centre studies^13–23^ solely investigated the relationship between abdominal ultrasound (US) findings and development of surgical NEC, two of which did so prospectively^13,23^. Study size varied with the smallest containing 15 infants whilst the largest had 158 participants. US at diagnosis of NEC was evaluated in all studies with one method describing subsequent use when there was uncertainty about decision making.^20^ Most studies reported signs seen on US associated with surgical NEC or surgical NEC and death. These included complex fluid collection^19^, portal venous gas^13,14^, pneumatosis^14^, pneumoperitoneum^22^, bowel dilation^21,22^, intestinal wall thickening^21,22^, ascites^21,22^, bowel wall thinning^13^ focal absence of peristalsis^13^ and absent bowel wall perfusion^13^. Other studies reported overall effectiveness of US with AUC as high as 0.917^20^ (Supplementary Table 6). All studies advocated the use of US in NEC and most acknowledged that this method is operator dependent and requires an experienced sonographer to provide accurate results. Meta-analysis was undertaken to determine which features on US were most predictive of surgical NEC (Table 2a) or surgical NEC and death (Table 2b). There were 16 studies^13–23,28,51,66–68^ that described features of US which were synthesised. The features most highly associated with identifying surgical NEC were absent bowel wall perfusion (OR 142.89 [95% CI 15.65–1305.00]) followed by pneumoperitoneum (OR 43.42 [95% CI 7.24–260.25]) then absent peristalsis (OR 18.56 [95% CI 1.29–266.89]). For identifying surgical NEC or death the most highly associated features were complex fluid collection (OR 15.96 [95% CI 6.53–38.98]), pneumoperitoneum (OR 11.00 [95% CI 3.03–39.94]) and absent bowel wall perfusion (OR 6.99 [95% CI 2.06–23.76]).

**Table 2:** a Meta-analysis of abdominal ultrasound (US) features predictive of surgical NEC. b Meta-analysis of abdominal ultrasound (US) features predictive of surgical NEC or death.

	Studies	Infants (*n*)	Odds ratio (95% CI)
*Absent bowel wall perfusion*	2	84	142.89 (15.65–1305.00)
*Pneumoperitoneum*	2	138	43.42 (7.24–260.25)
*Absent peristalsis*	2	293	18.56 (1.29–266.89)
*Focal fluid collection*	4	203	15.46 (4.81–49.66)
*Complex fluid collection*	4	203	12.36 (4.96–30.81)
*Thickened bowel wall*	6	644	4.72 (1.17–19.06)
*Thinned bowel wall*	5	460	4.26 (0.46–39.37)
*Intramural gas*	7	671	3.29 (1.35–7.99)
*Portal venous gas*	7	502	3.00 (1.15–7.80)
*Increased bowel wall perfusion*	4	222	2.51 (0.18–35.55)
*Bowel wall echogenicity*	1	83	2.36 (0.62–9.01)
*Simple free fluid*	4	377	1.50 (0.36–6.30)
*Dilated bowel*	1	84	0.66 (0.21–2.07)

	Studies	Infants (*n*)	Odds ratio (95% CI)
*Complex fluid collection*	4	180	15.96 (6.53–38.98)
*Pneumoperitoneum*	7	586	11.00 (3.03–39.94)
*Absent bowel wall perfusion*	3	120	6.99 (2.06–23.76)
*Focal fluid collection*	5	336	6.72 (1.37–33.09)
*Bowel wall echogenicity*	3	121	6.00 (1.74–20.63)
*Absent peristalsis*	5	390	5.46 (1.60–18.69)
*Thinned bowel wall*	5	266	4.08 (1.08–15.37)
*Thickened bowel wall*	8	611	3.87 (2.61–5.75)
*Dilated bowel*	3	305	3.81 (2.21–6.55)
*Portal venous gas*	8	611	3.70 (1.62–8.46)
*Increased bowel wall perfusion*	4	180	3.44 (0.58–20.19)
*Simple free fluid*	8	611	2.75 (0.73–10.32)
*Intramural gas*	8	611	2.35 (1.17–4.72)

Odds ratio represents odds of surgical NEC (2a) or surgical NEC or death (2b) versus medical NEC if US feature is present. Ordered by odds ratios.

#### Abdominal ultrasound and radiograph

Three single centre studies^66–68^ used features on abdominal US in combination with features on abdominal radiograph (AXR). One of these methods^67^ used a scoring system for features identified on either US or AXR (Supplementary Table 5) with the presence of 2 features taken as a positive test. This was associated with a sensitivity and specificity of 75.9% and 68.5% respectively (Supplementary Table 6). The authors concluded that both these imaging modalities should be used in NEC without pneumoperitoneum to decide whether surgery is indicated.

#### Abdominal radiograph

AXR use as an independent method was reported in seven single centre retrospective studies^15,28,69–73^. Various features and signs from the AXR were used. Zvizdic et al.^71^ reported that bowel wall diameter related to vertebral landmarks was greater in those that subsequently required surgery. Leonard et al.^69^ found that a fixed loop on AXR was seen more often in those that required surgery or died. Muller et al.^70^ found that the presence of a fixed loop on AXR had a sensitivity and specificity of 82% and 37% respectively for identifying surgical NEC. Other studies^15,28,72,73^ used a scoring system of features to determine severity of disease. The Duke Abdominal Assessment Scale (DAAS) was used in three studies^28,72,73^ which reported various levels of effectiveness. Two studies concluded that the DAAS is useful in predicting surgical NEC however one study^28^, which was the only one using a testing cohort, felt that it was a poor method and US was more effective.

#### Other imaging methods

Other methods explored in single centre studies were radionucleotide scan^74^, computer tomography^75^ and magnetic resonance imaging^76^. These were relatively small exploratory studies however they were effective (Supplementary Table 6) and authors conclude that they might be useful in NEC pending further evaluation.

### Invasive methods

There were nine reports identified of an invasive method, of these three were unique (Supplementary Table 7).

#### Laparoscopy

Laparoscopy was reported in five single centre studies^7,39–42^ and exact details of this technique varied. Some studies reported use of gasless laparoscopy, others with standard creation of pneumoperitoneum with carbon dioxide, and one study used fluorescein alongside laparoscopy to assess bowel perfusion (Supplementary Table 7). In these studies the two groups of infants were those who had findings at laparoscopy requiring conversion to laparotomy or drain insertion, or those with findings at laparoscopy that allowed continuation of medical management without any therapeutic surgical interventions. This method was reported to be highly effective at identifying those who did and did not require subsequent laparotomy, with measures of test effectiveness of 100% across all studies that reported these (Supplementary Table 8) however authors accepted it is very invasive and unlikely to be acceptable to use in many babies.

#### Paracentesis

Paracentesis was reported in three single centre retrospective studies^77–79^ all of which were relatively small. Various features of the paracentesis were used as a reference test in each study. These were neutrophil ratio of fluid^77^, brown colour or bacteria on smear of fluid^78^ or unspecified^79^. This method was reported to be effective at identifying surgical NEC with sensitivities and specificities above 90% in all three reported studies.

#### Intravesical pressure

Intravesical pressure was reported in one study^80^ and a threshold of 6.25 mmHg was found to be predictive of surgical NEC with a sensitivity and specificity of 100% and 75% respectively however this was not validated in a separate cohort of infants.

## Discussion

This study has identified and reported multiple existing methods within the published literature that prognosticate infants with NEC to either medical or surgical disease. Overall we identified 114 methods of which 63 are unique. The majority are scoring systems consisting of multiple clinical, biochemical, and radiological elements. Whilst several methods that have been identified show promise for clinical utility, most methods have not been validated, either internally or externally. Concerningly, a small number of studies have been published without sufficient detail in the methodology of the study to allow reuse of their reported test for further validation in clinical practice. This mainly applies to studies that used machine learning methodology.

There were several methods identified that appear useful and provide sufficient detail in the methodology to externally validate effectiveness and also implement into clinical practice. One of these is the MD7 score which was the most frequently identified clinical scoring system^5,24–29^ and appears to be an effective method of identifying those with surgical NEC without pneumoperitoneum. One important advantage of this test is that it uses readily available data without the need for complicated calculations and includes many elements used by clinicians already when assessing whether an infant with NEC should have surgery. It is therefore highly applicable and likely feasible to implement in developed healthcare settings. Further it has sensitivity and specificity both in excess of 70% (although we acknowledge meta-analysis was limited in this review as only 2 studies could be included due to heterogeneity between studies). The MD7 score has been previously evaluated by Tepas et al. who found that use of the MD7 score was associated with better outcome (death or parenteral nutrition requirement) compared to a neonatal unit that did not use this score.^24^ In contrast to the MD7 score, a limitation of some of the reported scoring systems identified was how many features contribute to the score; this was as high as 49 features in one.^44^ Use of such a method is unlikely to be feasible in day to day practice.

Most of the biomarkers identified are measures of systemic inflammation, although the most frequently evaluated biomarker, i-FABP, is specific to enterocytes of the small bowel and is found in increased concentrations in the circulation where there is ischaemia, inflammation or mucosal damage.^81,82^ Both plasma and urine i-FABP appear effective at identifying surgical NEC but unfortunately, robust conclusions cannot be drawn from a quantitative synthesis of these studies as thresholds for a positive test varied. A major drawback of i-FABP is that the current time taken to process this test is too long to be useful in a disease that rapidly progresses. A physiological biomarker that is able to be measured in real time is heart rate and heart rate characteristic (HRC) index is a non-invasive measure of heart rate variability.^63^ Initially developed for neonatal sepsis, the HRC index incorporates decreased heart rate variability and transient decelerations to generate the probability of an infant developing sepsis in the subsequent 24 h, using logistic regression. The identified report of its use in surgical NEC is promising with prediction of surgical NEC, and differentiation from medical NEC, even prior to diagnosis of disease.^63^ Importantly this method is highly applicable since HRC index algorithms can be incorporated into bedside monitors.

Meta-analysis found several abdominal US features which were highly suggestive of surgical NEC and/or death suggesting that US is a promising modality for evaluation of infants with NEC. Our findings are similar to those reported in a previous meta-analysis published in 2017.^6^ This technique appears effective on neonatal units with skilled and experienced sonographers however it does not provide a continuous measure as it is limited to the time at which the examination is undertaken. Studies to date have focussed on individual features on US and their association with disease progression. Some have explored combining these features with varied success.^15,16,18,20,23^ What is yet to be seen is how routine and more widespread use of US impacts clinical outcome and further studies should explore this healthcare technology. Diagnostic laparoscopy, to determine requirement for laparotomy, appeared effective in a highly selective group of infants where there was enough uncertainty about whether surgery was indicated or not to proceed with this invasive examination.^7,41,42^ A study from France comparing those with NEC who had laparoscopy initially, versus laparotomy, found that those who had laparoscopy underwent definitive surgery earlier, had reduced inflammatory response and a lower rate of intestinal stricture.^83^ The reduced time to surgery is particularly interesting here as it would suggest that clinicians are more willing to undertake a less invasive measure such as a laparoscopy and therefore have a lower threshold for intervention, rather than delaying this.^84^ Laparoscopy may not be feasible in the smallest of infants encountered with NEC, but in the absence of an effective non-invasive method, it has its utility where there is uncertainty.

This systematic review is the first of its kind to comprehensively summarise and evaluate multiple reported methods for identifying the need for surgical intervention in babies with NEC. This has included studies in all languages. Meta-analysis has been possible for two of these methods allowing pooled estimates of their effectiveness. As with all studies, there are limitations. Different definitions of both NEC and surgical NEC were used and there was no consensus between studies on whether infants that died of NEC met the definition or surgical NEC or not. Some studies excluded those with pneumoperitoneum whereas others included this group of infants with NEC. Including these may allow earlier identification of impending perforation, allowing intervention prior to this occurring, however some may argue that this since pneumoperitoneum is a clear indication for surgical intervention, identification of need for surgery in this group of infants is superfluous. Any systematic review is limited by the quality of the included studies, many included here were single centre retrospective datasets without internal nor external validation. However, there was no shortage of large multicentre studies included within this work. Positively, many studies provided reports of the same method however most reported different thresholds for positive tests meaning meta-analysis was not feasible.

As well as test effectiveness, there are a number of important considerations when determining whether a method can be applied to clinical practice, specifically for this population of patients. Some studies have considered this whilst others have not. Any useful method in NEC must provide a result within hours, it should be simple enough that clinicians can readily use it without the burden of pooling many different data from different sources, the method should not cause harm to the baby and it is more likely to be adopted if the methodology is understandable to clinicians. Furthermore, none of the included studies revealed routine use of any of the methods identified.

In conclusion, several methods had high measures of test effectiveness (i.e., sensitivity, specificity, and AUC) for distinguishing surgical from medical NEC. The next stage in this work is to further evaluate those methods that hold promise in a large external dataset (i.e., external validation) to truly understand which, if any, have potential for widespread use in practice. The methods we would recommend for future evaluation based on an overall assessment of reported effectiveness, repeatability, feasibility, and acceptability are the MD7 score^5^ (given its simplicity and reported effectiveness in seven studies), the composite score by Khalak et al.^27^ (due to the prospective development of this test and reported effectiveness at several time points), the clinical score by Kang et al.^50^ (an easily applicable score developed on a multicentre dataset with high reported effectiveness), the coagulation profile and full blood count scores by Feng et al.^85,86^ (effective and use readily available routinely collected data) and plasma CRP trajectory^35^ (accounts for disease trajectory and appears effective in a small cohort of infants). Other methods which do not rely on routinely available data but appear to hold promise, and therefore warrant further investigation include abdominal US and heart rate variability index. Ultimately, prospective clinical evaluation of a number of these methods is required to determine impact of their use on outcomes in NEC.

## Supplementary information

Checklist

Supplementary information

Supplementary Tables

## Data Availability

The datasets generated during and/or analysed during the current study are available from the corresponding author on reasonable request.
