# Supplementary material for: Methods of identifying surgical Necrotizing Enterocolitis—a systematic review and meta-analysis
Source: Pediatr Res. 2024 Jun 7;97(1):45–55. doi: 10.1038/s41390-024-03292-3 (PMC11798840; doi:10.1038/s41390-024-03292-3)
Supplement: Supplementary file 2 — Supplementary information [file 41390_2024_3292_MOESM2_ESM.pdf]

Systematic review search strategy

Medline

Ovid MEDLINE(R) and Epub Ahead of Print, In-Process, In-Data-Review & Other Non-Indexed Citations, Daily and Versions <1946 to October 20, 2022>

1	*Enterocolitis, Necrotizing/
2	surgery.mp.
3	surgical.mp.
4	*Biomarkers/
5	identification.mp.
6	test.mp.
7	*Diagnosis/
8	severity.mp
9	1 and 2
10	1 and 3
11	9 or 10
12	4 or 5 or 6 or 7 or 8
13	11 and 12

Embase

(((Necrotising Enterocolitis) AND ((surgical[Title/Abstract]) OR (surgery[Title/Abstract]))) AND ((biomarker) OR (identification[Title/Abstract]) OR (test[Title/Abstract]) OR (diagnosis) OR (severity[Title/Abstract])) Sort by: Most Recent

("necrotising enterocolitis"[All Fields] OR "enterocolitis, necrotizing"[MeSH Terms] OR ("enterocolitis"[All Fields] AND "necrotizing"[All Fields]) OR "necrotizing enterocolitis"[All Fields] OR ("necrotizing"[All Fields] AND "enterocolitis"[All Fields])) AND ("surgical"[Title/Abstract] OR "surgery"[Title/Abstract]) AND ("biomarker s"[All Fields] OR "biomarkers"[MeSH Terms] OR "biomarkers"[All Fields] OR "biomarker"[All Fields] OR "identification"[Title/Abstract] OR

"test"[Title/Abstract] OR ("diagnosable"[All Fields] OR "diagnosi"[All Fields] OR "diagnosis"[MeSH Terms] OR "diagnosis"[All Fields] OR "diagnose"[All Fields] OR "diagnosed"[All Fields] OR "diagnoses"[All Fields] OR "diagnosing"[All Fields] OR "diagnosis"[MeSH Subheading]) OR "severity"[Title/Abstract])

Translations

Necrotising Enterocolitis: "necrotising enterocolitis"[All Fields] OR "enterocolitis, necrotizing"[MeSH Terms] OR ("enterocolitis"[All Fields] AND "necrotizing"[All Fields]) OR "necrotizing enterocolitis"[All Fields] OR ("necrotizing"[All Fields] AND "enterocolitis"[All Fields])

biomarker: "biomarker's"[All Fields] OR "biomarkers"[MeSH Terms] OR "biomarkers"[All Fields] OR "biomarker"[All Fields]

diagnosis: "diagnosable"[All Fields] OR "diagnosi"[All Fields] OR "diagnosis"[MeSH Terms] OR "diagnosis"[All Fields] OR "diagnose"[All Fields] OR "diagnosed"[All Fields] OR "diagnoses"[All Fields] OR "diagnosing"[All Fields] OR "diagnosis"[Subheading]

Scopus

(ALL (necrotising AND enterocolitis) AND (ABS (surgical) OR ABS (surgery))) AND (ALL (biomarker) OR ABS (identification) OR ABS (test) OR ALL (diagnosis) OR ABS (severity))

Web of Science

1 ALL Enterocolitis, Necrotizing/

2 surgery.AB

3 surgical.AB

4 ALL Biomarkers/

5 identification.AB

6 test.AB

7 ALL Diagnosis/

8 severity.AB

9 1 and 2

10 1 and 3

11 9 or 10

12 4 or 5 or 6 or 7 or 8

13 11 and 12

Cochrane library

#1 MeSH descriptor: [Enterocolitis, Necrotizing] explode all trees

#2 (surgical):ab

#3 (surgery):ab

#4 (biomarker):ti,ab,kw

#5 (identification):ab

#6 (test):ab

#7 (diagnosis):ti,ab,kw

#8 (severity):ab

#9 #1 AND #2

#10 #1 AND #3

#11 #9 OR #10

#12 #4 OR #5 OR #6 OR #7 OR #8

#13 #11 AND #12
