# Supplementary material for: Methods of identifying surgical Necrotizing Enterocolitis—a systematic review and meta-analysis
Source: Pediatr Res. 2024 Jun 7;97(1):45–55. doi: 10.1038/s41390-024-03292-3 (PMC11798840; doi:10.1038/s41390-024-03292-3)
Supplement: Supplementary file 3 — Supplementary Tables [file 41390_2024_3292_MOESM3_ESM.pdf]

Supplementary table 1 - study and test characteristics of methods reporting a clinical scoring system.

Study	***Country***	***Years***	***Multicentre***	***Prospective***	***Medical (n)***	***Surgical (n)***	***Inclusion criteria***	***Reference standard***	***Index test summary***	***Repeatable***	***Threshold (points or features)***	***Timing***	***Train and test cohort***
Tepas 2010[5]	USA	2005 - 2008	No	No	16	19	Clinical and radiological features of NEC	Surgery	MD7 score	Yes	≥3	Diagnosis	No
Tepas 2010[24]	USA	2005 - 2008	Yes	Yes	41	65	Clinical and radiological features of NEC	Surgery	MD7 score	Yes	≥3	Diagnosis	Yes
Ibanez 2012[25]	Spain	2002 - 2008	No	No	64	35	Bell's II and III	Surgery or death from NEC	MD7 score	Yes	≥3	Diagnosis	Test
Arni 2017[26]	Switzerland	1991 - 2011	No	No	27	12	Bell's II and III	Surgery	MD7 score	Yes	≥3	Diagnosis	Test
Khalak 2018[27]	USA	2014 - 2016	Yes	Yes	60	35	Bell's II and III	Surgery or death	MD7 score	Yes	≥3	Diagnosis	No
Yu 2022[28]	China	2015 - 2019	No	No	143	41	Bell's II and III	Surgery	MD7 score	Yes	≥3	Diagnosis	Test
Fijas 2022[29]	USA	2010 - 2019	No	No	44	20	Clinical and radiological features of NEC	Surgery	MD7 score	Yes	2	Diagnosis	Test

Study	Country	Years	Multicentre	Prospective	Medical (n)	Surgical (n)	Inclusion criteria	Reference standard	Index test summary	Repeatable	Threshold (points or features)	Timing	Train and test cohort
Yu 2022[28]	China	2015 - 2019	No	No	143	41	Bell's II and III	Surgery	MD7 score and DAAS	Yes	≥7 (DAAS) and ≥3 (MD7)	Diagnosis	No
Khalak 2018[27]	USA	2014 - 2016	Yes	Yes	60	35	Bell's II and III	Surgery or death	MD7 score and 7 feature clinical score	Yes	NS	Diagnosis	No
Ibanez 2012[25]	Spain	2002 - 2008	No	No	64	35	Bell's II and III	Surgery or death from NEC	SNAPPE-II	Yes	15.5	Diagnosis	Test
Lin 2013[43]	China	2001 - 2011	No	No	42	20	Bell's I, II and III	Surgery	SNAPPE-II	Yes	22	Diagnosis	No
Fijas 2022[29]	USA	2010 - 2019	No	No	44	20	Clinical and radiological features of NEC	Surgery	SNAPPE-II	Yes	29.04	Diagnosis	Test
Lewis 2022[87]	USA	2008 - 2020	Yes	No	145	114	Bell's II and III	Surgery or death	nSOFA score	Yes	≥4	NEC diagnosis - 6 hours pre to 12 hours post	No

Study	*Country*	*Years*	*Multicentre*	*Prospective*	*Medical (n)*	*Surgical (n)*	*Inclusion criteria*	*Reference standard*	*Index test summary*	*Repeatable*	*Threshold (points or features)*	*Timing*	*Train and test cohort*
Lin 2013[43]	China	2001 - 2011	No	No	42	20	Bell's I, II and III	Surgery	SNAP-II	Yes	22	Diagnosis	No
Gao 2021[44]	China	2011 - 2020	No	No	268	111	Bell's I, II and III	Surgery	49 feature clinical and radiological score	No	0.8	Diagnosis	Yes
Irles 2018[88]	Mexico	2015 - 2017	No	No	23	26	Bell's II and III	Perforation from NEC	35 feature clinical and radiological score	No	NS	Birth	Yes
Sylvester 2014[48]	USA	2007 - 2012	Yes	Yes	44	20	Clinical concern of NEC	Surgery or death from NEC	30 feature clinical score	No	-	Suspected NEC	Yes
Sylvester 2014[48]	USA	NS	Yes	Yes	345	140	Clinical concern of NEC	Surgery or death from NEC	27 feature clinical score	No	NS	Suspected NEC	Yes
Rao 2022[49]	China	2016 - 2021	No	No	70	36	Bell's II and III	Surgery with necrosis or perforation	21 feature clinical and radiological score	Yes	>3.5	NEC diagnosis - up to 48 hours post	No

Study	*Country*	*Years*	*Multicentre*	*Prospective*	*Medical (n)*	*Surgical (n)*	*Inclusion criteria*	*Reference standard*	*Index test summary*	*Repeatable*	*Threshold (points or features)*	*Timing*	*Train and test cohort*
Song 2022[46]	China	2015 - 2021	No	No	205	91	Bell's II and III	Surgery or death	19 feature clinical score	No	NS	Diagnosis	Yes
Arni 2017[26]	Switzerland	1991 - 2011	No	No	31	26	Bell's II and III	Surgery	13 feature clinical and radiological score	Yes	≥3	Diagnosis	Test
Munaco 2015[89]	USA	2001 - 2010	No	No	75	122	Bell's II and III	Surgery	13 feature clinical and radiological score	Yes	≥3	Diagnosis	No
Caro 1989[90]	Chile	1984 - 1987	No	Yes	12	6	Bell's I, II and III	NS	10 feature clinical and radiological score	Yes	15	Diagnosis	No
Ji 2014[47]	China and USA	2003 - 2011	Yes	Yes	344	140	Bell's I, II and IIIa	Surgery with confirmed NEC	10 feature clinical and radiological score	No	NS	Diagnosis	Yes
Kang 2022[50]	China	2018 - 2020	Yes	No	78	57	Bell's II and III	Surgery	8 feature clinical score	Yes	5.46	Diagnosis	No

Study	Country	Years	Multicentre	Prospective	Medical (n)	Surgical (n)	Inclusion criteria	Reference standard	Index test summary	Repeatable	Threshold (points or features)	Timing	Train and test cohort
Khalak 2018[27]	USA	2014 - 2016	Yes	Yes	60	35	Bell's II and III	Surgery or death	7 feature clinical score	Yes	≥3	Diagnosis	No
Sylvester 2014[91]	USA	2007 - 2012	Yes	Yes	59	26	Bell's II and III	Surgery	6 feature urine protein panel	Yes	Mean ratio up-regulated to down-regulated assays	Diagnosis	Yes
Gephart 2017[92]	USA	2008 - 2013	Yes	No	19	44	Clinical concern of NEC	Surgery	6 feature clinical score	Yes	6	NEC diagnosis - up to 36 hours pre	No
German 1979[93]	USA	1975 - 1976	No	No	29	11	Clinical concern of NEC	Surgery	5 feature clinical and radiological score	Yes	>10	Diagnosis	Yes
Pantalone 2021[94]	USA	2010 - 2019	No	Yes	116	61	Bell's II and III	Surgery	5 feature clinical score	Yes	As per decision tree	Diagnosis	No
Garcia 2019[95]	Spain	2003 - 2015	No	No	77	47	Bell's I, II and III	Surgery	4 feature clinical score	No	2.5	Diagnosis	No

Study	***Country***	***Years***	***Multicentre***	***Prospective***	***Medical (n)***	***Surgical (n)***	***Inclusion criteria***	***Reference standard***	***Index test summary***	***Repeatable***	***Threshold (points or features)***	***Timing***	***Train and test cohort***
Lazow 2021[51]	USA	2009 - 2018	Yes	No	69	14	Clinical concern of NEC	Surgery inc. delayed for stricture	4 feature clinical and radiological score	Yes	3 or 4	Diagnosis	No
Gupta 1994[96]	USA	1983 - 1990	No	No	19	23	Bell's II and III	Surgery	4 feature clinical score	Yes	≥3	NEC diagnosis - 4 to 12 hours post	No
Diez 2022[97]	Germany	2010 - 2020	No	No	57	31	Bell's I, II and III	Bell's III	3 feature clinical and radiological score	Yes	≥3	Diagnosis	No
Liu 2022[98]	China	2019 - 2020	No	Yes	26	23	Bell's II and III	Surgery	3 feature clinical score	Yes	≥2	Diagnosis	No
Ng 2013[99]	Hong Kong	NS	No	Yes	8	12	Bell's II and III	Surgery	3 feature clinical score	Yes	4.5	Diagnosis	No
Feng 2022[86]	China	2019 - 2020	No	No	68	46	Clinical and radiological features of NEC	Surgery inc. delayed for stricture	3 feature coagulation profile score	Yes	1 of 3 criteria	Diagnosis	No

Study	*Country*	*Years*	*Multicentre*	*Prospective*	*Medical (n)*	*Surgical (n)*	*Inclusion criteria*	*Reference standard*	*Index test summary*	*Repeatable*	*Threshold (points or features)*	*Timing*	*Train and test cohort*
Feng 2022[85]	China	2019 - 2020	No	No	82	49	Bell's II and III	Surgery inc. delayed for stricture	3 feature full blood count score	Yes	>235.85	Diagnosis	No
Mohd Amin 2021[52]	UK	2009 - 2018	No	No	107	84	Clinical and radiological features of NEC	Surgery	2 feature clinical score	Yes	≥2	Day 1 of diagnosis	No
Reisinger 2014[53]	Netherlands	2008 - 2010	Yes	Yes	12	17	Bell's II and III	Surgery or death from NEC	2 feature clinical score	No	Platelet count–256(SAA)= 159.3	Diagnosis or one day prior to surgery[£]	No
Buras 1986[54]	USA	1979 - 1984	No	No	45	47	Clinical and radiological features of NEC	Surgery	2 feature clinical and radiological score	Yes	2	NEC diagnosis onwards	No
Pourcyrous 2005[55]	USA	1998	No	Yes	48	7	Bell's II and III	Bell's III	2 feature clinical and radiological score	No	NS	NS	No
Yu 2018[56]	China	2015 - 2018	No	No	43	41	Bell's II and III	Surgery	2 feature full blood count score	Yes	0.55	Decision to operate or diagnosis &	Yes

Study	***Country***	***Years***	***Multicentre***	***Prospective***	***Medical (n)***	***Surgical (n)***	***Inclusion criteria***	***Reference standard***	***Index test summary***	***Repeatable***	***Threshold (points or features)***	***Timing***	***Train and test cohort***
												36 hours (medical)	
Qi 2022[45]	China	NS	No	No	45 total		Clinical concern of NEC	NS	NS	No	NS	NS	No

Table shows study demographics and index test features. n = number, MD7 = metabolic derangement 7 score, USA = United States of America, NEC = necrotising enterocolitis, DAAS = duke abdominal assessment scale, SNAPPE-II = Score for Neonatal Acute Physiology Perinatal Extension, nSOFA = Neonatal Sequential Organ Failure Assessment, SNAP II = Score for Neonatal Acute Physiology, UK = United Kingdom, £ = diagnosis if operation same day (or death) or one day prior to surgery if operation after day of diagnosis, SAA = serum amyloid A.

Supplementary table 2 - effectiveness of clinical scoring system methods.

Study	*Index test summary*	*Threshold (points or features)*	*Timing*	*Timing of reapplication*	**Test effectiveness**					
					Sens (%)	*Spec (%)*	*PPV (%)*	*NPV (%)*	*AUC (%)*	*Reapplied test*
Tepas 2010[5]	MD7 score	≥3	Diagnosis	Yes, NS	Median metabolic derangement score of 3 or more seen in cases with a bad outcome (death or ongoing parenteral nutrition)					
Tepas 2010[24]	MD7 score	≥3	Diagnosis	Yes, NS	Poor outcome (death or ongoing parenteral nutrition) seen more often in unit where MD7 score not used					
Ibanez 2012[25]	MD7 score	≥3	Diagnosis	Soonest of clinical deterioration or 36 hours post diagnosis					0.59	AUC = 0.64
Arni 2017[26]	MD7 score	≥3	Diagnosis	No	92	59	50	94		
Khalak 2018[27]	MD7 score	≥3	Diagnosis	8 to 12 hours, 12 to 36 hours and 36 to 48 hours					0.74	
Yu 2022[28]	MD7 score	≥3	Diagnosis	No	53.8	83.1	47.7	86.3		
Fijas 2022[29]	MD7 score	2	Diagnosis	No	70	66			0.77	

Study	*Index test summary*	*Threshold (points or features)*	*Timing*	*Timing of reapplication*	*Sens (%)*	*Spec (%)*	*PPV (%)*	*NPV (%)*	*AUC (%)*	*Reapplied test*
Yu 2022[28]	MD7 score and DAAS	≥7 (DAAS) and ≥3 (MD)	Diagnosis	No	12.8	100	100	80		
Khalak 2018[27]	MD7 and 7 feature clinical score	NS	Diagnosis	8 to 12 hours, 12 to 36 hours and 36 to 48 hours					0.89	
Ibanez 2012[25]	SNAPPE-II score	15.5	Diagnosis	Soonest of clinical deterioration or 36 hours post diagnosis					0.69	AUC = 0.67
Lin 2013[43]	SNAPPE-II score	22	Diagnosis	No					0.71	
Fijas 2022[29]	SNAPPE-II score	29.04	Diagnosis	No	70	60			0.71	
Lewis 2022[87]	nSOFA score	≥4	NEC diagnosis - 6 hours pre to 12 hours post	Every 6 hours	67	90	84	78	0.84	
Lin 2013[43]	SNAP-II score	22	Diagnosis	No					0.75	

Study	***Index test summary***	***Threshold (points or features)***	***Timing***	***Timing of reapplication***	***Sens (%)***	***Spec (%)***	***PPV (%)***	***NPV (%)***	***AUC (%)***	***Reapplied test***
Gao 2021[44]	49 feature clinical and radiological score	0.8	Diagnosis	No	85	95.4	97.1	90.6	0.94	
Irles 2018[88]	35 feature clinical and radiological score	NS	Birth	24 hours pre-perforation			R^2 =0.976			R^2=0.980
Sylvester 2014[48]	30 feature clinical score	-	Suspected NEC	No	100	100	100	100		
Sylvester 2014[48]	27 feature clinical score	NS	Suspected NEC	No					0.894 training and 0.817 test	
Rao 2022[49]	21 feature clinical and radiological score	>3.5	NEC diagnosis - up to 48 hours post	No	97.2	91.4			0.98	
Song 2022[46]	19 feature clinical score	NS	Diagnosis	No		68.9	93.3		0.918	

Study	Index test summary	Threshold (points or features)	Timing	Timing of reapplication	Sens (%)	Spec (%)	PPV (%)	NPV (%)	AUC (%)	Reapplied test
Arni 2017[26]	13 feature clinical and radiological score	≥3	Diagnosis	No	100	13	49	100		
Munaco 2015[89]	13 feature clinical and radiological score	≥3	Diagnosis	Yes, NS	Presence of 3 features significantly associated with surgical intervention.					
Caro 1989[90]	10 feature clinical and radiological score	15	Diagnosis	8 to 12 hours	A score of 15 or more associated with surgical intervention.					
Ji 2014[47]	10 feature clinical and radiological score	NS	Diagnosis	No			93.8		0.85	
Kang 2022[50]	8 feature clinical score	5.46	Diagnosis	No	97	92			0.98	
Khalak 2018[27]	7 feature clinical score	≥3	Diagnosis	8 to 12 hours, 12 to 36 hours and 36 to 48 hours	88	80	70	92	0.89	

Study	Index test summary	Threshold (points or features)	Timing	Timing of reapplication	Sens (%)	Spec (%)	PPV (%)	NPV (%)	AUC (%)	Reapplied test
Sylvester 2014[91]	6 feature urine protein panel	Mean ratio up-regulated to down-regulated assays	Diagnosis	No	89	90			98.4	
Gephart 2017[92]	6 feature clinical score	6	NEC diagnosis - up to 36 hours pre	24 hours, 12 hours and 6hr pre diagnosis, diagnosis	Highest severity associated with more abdominal signs leading up to and at diagnosis of NEC.					
German 1979[93]	5 feature clinical and radiological score	>10	Diagnosis	No	90.9	100	100	96.7		
Pantalone 2021[94]	5 feature clinical score	As per decision tree	Diagnosis	No	36.7	81.9	51.6	71.1	0.759	
Garcia 2019[95]	4 feature clinical score	2.5	Diagnosis	No	81.8	76.7			0.829	
Lazow 2021[51]	4 feature clinical and radiological score	3 or 4	Diagnosis	No	94.9 (3) or 99.7 (4)				0.937	

Study	*Index test summary*	*Threshold (points or features)*	*Timing*	*Timing of reapplication*	*Sens (%)*	*Spec (%)*	*PPV (%)*	*NPV (%)*	*AUC (%)*	*Reapplied test*
Gupta 1994[96]	4 feature clinical score	≥3	NEC diagnosis - 4 to 12 hours post	12 to 24 hours	64	100	100			Sens = 55, Spec = 89, PPV = 70, NPV = 80
Diez 2022[97]	3 feature clinical and radiological score	≥3	Diagnosis	Every 24 hours	68	69			0.78	
Liu 2022[98]	3 feature clinical score	≥2	Diagnosis	No	82.6	92.3			0.943	
Ng 2013[99]	3 feature clinical score	4.5	Diagnosis	No	83	100				
Feng 2022[86]	3 feature coagulation profile score	1 of 3 criteria	Diagnosis	No	82.6	91.2	86.4	88.6	0.869	
Feng 2022[85]	3 feature full blood count score	>235.85	Diagnosis	No	87.76	73.17	66.15	90.91	0.833	
Mohd Amin 2021[52]	2 feature clinical score	≥2	Day 1 of diagnosis	Day 2 and 3 (threshold ≥3)	43.9	76.1			0.6	Sens = 76.4, Spec = 71.9,

Study	*Index test summary*	*Threshold (points or features)*	*Timing*	*Timing of reapplication*	*Sens (%)*	*Spec (%)*	*PPV (%)*	*NPV (%)*	*AUC (%)*	*Reapplied test*
										AUC = 0.72
Reisinger 2014[53]	2 feature clinical score	Platelet count–256(SAA)= 159.3	Diagnosis or one day prior to surgery£	No	94	83			0.93	
Buras 1986[54]	2 feature clinical and radiological score	2	NEC diagnosis onwards	Yes, NS	Persistent acidosis and portal venous gas associated with requiring surgery.					
Pourcyrous 2005[55]	2 feature clinical and radiological score	NS	NS	No		100				
Yu 2018[56]	2 feature full blood count score	0.55	Decision to operate or diagnosis + 36 hours (medical)	No	85	81			0.84	
Qi 2022[45]	Clinical and radiological - features not-specified	NS	NS	No					0.8	

Study	*Index test summary*	*Threshold (points or features)*	*Timing*	*Timing of reapplication*	*Sens (%)*	*Spec (%)*	*PPV (%)*	*NPV (%)*	*AUC (%)*	*Reapplied test*

Table shows effectiveness of clinical scoring systems at identifying surgical necrotising enterocolitis (NEC). Sens = sensitivity, spec = specificity, PPV = positive predictive value, NPV = negative predictive value, AUC = area under receiver operating characteristic curve, MD7 = metabolic derangement 7 score, DAAS = duke abdominal assessment scale, SNAPPE-II = Score for Neonatal Acute Physiology Perinatal Extension, nSOFA = Neonatal Sequential Organ Failure Assessment, SNAP II = Score for Neonatal Acute Physiology, £ = diagnosis if operation same day (or death) or one day prior to surgery if operation after day of diagnosis, SAA = serum amyloid A.

Supplementary table 3 - study and test characteristics of methods reporting a single biomarker.

Study	*Country*	*Years*	*Multicentre*	*Prospective*	*Medical (n)*	*Surgical (n)*	*Inclusion criteria*	*Reference standard*	*Index test summary*	*Repeatable*	*Threshold (points or features)*	*Timing*	*Train and test cohort*
Evennett 2010[31]	UK	2002 - 2003	No	Yes	4	12	Bell's II and III	Pan-intestinal NEC and focal NEC	Urinary intestinal fatty acid–binding protein to creatinine ratio	Yes	NS	Diagnosis	No
Thuijls 2010[8]	Netherlands	2005 - 2008	No	Yes	7	7	Bell's II and III	Surgery or death from NEC	Urinary intestinal fatty acid–binding protein to creatinine ratio	No	6.38 pg/nmol	Diagnosis	No
Schurink 2015[33]	Netherlands	2010 - 2012	No	Yes	11	11	Bell's II and III	Bell's III	Urinary intestinal fatty acid–binding protein to creatinine ratio	Yes	687 ng/mL	Diagnosis	No
Heida 2015[32]	Netherlands	2010 - 2012	Yes	Yes	0	19	Surgery for NEC	Surgical resection length	Urinary and plasma intestinal fatty acid–binding protein	Yes	NS	Diagnosis	No

Study	*Country*	*Years*	*Multicentre*	*Prospective*	*Medical (n)*	*Surgical (n)*	*Inclusion criteria*	*Reference standard*	*Index test summary*	*Repeatable*	*Threshold (points or features)*	*Timing*	*Train and test cohort*
El-Abd Ahmed 2020[30]	Egypt	2018	No	Yes	25	10	Bell's II and IIIA	Bell's IIIa	Urinary intestinal fatty acid–binding protein to creatinine ratio	Yes	> 2.93 ng/g	Diagnosis	No
Schurink 2015[33]	Netherlands	2010 - 2012	No	Yes	11	11	Bell's II and III	Bell's III	Plasma Intestinal fatty acid-binding protein	Yes	53 ng/mL	Diagnosis	No
El-Abd Ahmed 2020[30]	Egypt	2018	No	Yes	25	10	Bell's II and IIIA	Bell's IIIa	Plasma Intestinal fatty acid-binding protein	Yes	3.24 ng/ml	Diagnosis	No
Tayman 2011[36]	Turkey	2009 - 2010	Yes	Yes	12	10	Bell's II and III	Surgery	Plasma CRP	Yes	NS	Diagnosis	No
Wang 2018[37]	China	2015 - 2018	No	No	124	18	Bell's I, II and III	Surgery	Plasma CRP	Yes	14.6 mg/l	Before treatment of NEC	No
Cakir 2020[34]	Turkey	2014 - 2017	No	Yes	9	8	Bell's II and III	Surgery	Plasma CRP	Yes	15.7 mg/l	Diagnosis	No

Study	Country	Years	Multicentre	Prospective	Medical (n)	Surgical (n)	Inclusion criteria	Reference standard	Index test summary	Repeatable	Threshold (points or features)	Timing	Train and test cohort
Yu 2022[38]	China	2015 - 2019	No	No	143	41	NEC as per 4th Edition of Practice of Neonatology	Surgery	Plasma CRP	Yes	18.5 mg/L	Diagnosis	No
Srinivasjois 2010[35]	Australia	2001 - 2006	No	No	6	31	Bell's II and III	Surgery or death from NEC	Plasma CRP change	Yes	≥390%	Diagnosis to 72hrs	No
Morecroft 1994[57]	UK	1992 - 1993	Yes	Yes	8	7	Bell's I, II and III	Bell's III	Plasma interleukin 6	Yes	NS	Diagnosis	No
Tayman 2011[36]	Turkey	2009 - 2010	Yes	Yes	12	10	Bell's II and III	Surgery	Serum interleukin 6	Yes	NS	Diagnosis	No
Wisgrill 2019[58]	Austria	2015 - 2017	No	No	12	12	Clinical and radiological features of NEC	Surgery	Serum interleukin 6	Yes	1440 pg/ml	Diagnosis	No
Cakir 2020[34]	Turkey	2014 - 2017	No	Yes	9	8	Bell's II and III	Surgery	Serum interleukin 6	Yes	107 pg/ml	Diagnosis	No
Ververidis 2001[60]	UK	1995 - 1998	No	No	10	49	Bell's II and III	Full thickness necrosis at surgery	Consecutive platelet counts	Yes	2 tests <100 x 10^9/L	Diagnosis onwards	No

Study	*Country*	*Years*	*Multicentre*	*Prospective*	*Medical (n)*	*Surgical (n)*	*Inclusion criteria*	*Reference standard*	*Index test summary*	*Repeatable*	*Threshold (points or features)*	*Timing*	*Train and test cohort*
Ververidis 2001[60]	UK	1995 - 1998	No	No	10	49	Bell's II and III	Full thickness necrosis at surgery	Platelet count trajectory	Yes	>150 x 10^9/L drop in 24 hours reaching ≤ 100 x 10^9/L	Diagnosis onwards	No
Kenton 2005[59]	USA	1997 - 2001	No	No	30	61	Bell's II and III	Surgery	Platelet count	Yes	100,000/mm^3	Diagnosis	No
Tayman 2011[36]	Turkey	2009 - 2010	Yes	Yes	12	10	Bell's II and III	Surgery	Serum amyloid A	Yes	NS	Diagnosis	No
Coufal 2020[61]	Czech Republic	2012 - 2014	No	Yes	11	9	Bell's I, II and III	Surgery	Serum amyloid A	Yes	NS	Diagnosis	No
Wang 2018[37]	China	2015 - 2018	No	No	124	18	Bell's I, II and III	Surgery	Plasma Procalcitonin	Yes	1.4 ng/mL	Before treatment of NEC	No
Yu 2022[38]	China	2015 - 2019	No	No	143	41	NEC as per 4th Edition of Practice of Neonatology	Surgery	Plasma Procalcitonin	Yes	1.825 ng/ml	Diagnosis	No

Study	*Country*	*Years*	*Multicentre*	*Prospective*	*Medical (n)*	*Surgical (n)*	*Inclusion criteria*	*Reference standard*	*Index test summary*	*Repeatable*	*Threshold (points or features)*	*Timing*	*Train and test cohort*
Stone 2013[62]	USA	2005 - 2010	Yes	No	64	33	Bell's II and III	Surgery within 7 days diagnosis	Heart rate characteristic index	Yes	NA	Continuous including prior to NEC diagnosis	No
van der Schyff 2018[63]	South Africa	2000 - 2014	No	No	13	32	Bell's II and III	Surgery with necrosis or perforation	Mean heart rate	Yes	NA	Continuous	No
Srinivasjois 2010[35]	Austra lia	2001 - 2006	No	No	6	31	Bell's II and III	Surgery or death from NEC	Lactate change	Yes	≥147%	Diagnosis to 48hrs	No
Yu 2022[38]	China	2015 - 2019	No	No	143	41	NEC as per 4th Edition of Practice of Neonatology	Surgery	Lactate	Yes	1.25 mmol/L	Diagnosis	No
Klein 1986[64]	USA	1982 - 1986	No	No	41	21	Clinical, radiological and histological features of NEC	Surgery	Red cell T-Cryptantigen activation	Yes	T-activated vs not	Diagnosis	No
Hall 2002[65]	UK	1991 - 2000	No	Yes	31	73	Bell's II and III	Bell's III	Red cell T-Cryptantigen activation	Yes	T-activated vs not	Diagnosis	No
Tayman 2011[36]	Turkey	2009 - 2010	Yes	Yes	12	10	Bell's II and III	Surgery	C5a	Yes	NS	Diagnosis	No

Study	*Country*	*Years*	*Multicentre*	*Prospective*	*Medical (n)*	*Surgical (n)*	*Inclusion criteria*	*Reference standard*	*Index test summary*	*Repeatable*	*Threshold (points or features)*	*Timing*	*Train and test cohort*
Cakir 2020[34]	Turkey	2014 - 2017	No	Yes	9	8	Bell's II and III	Surgery	Endocan	Yes	1114.65 ng/ml	Diagnosis	No
Yu 2022[38]	China	2015 - 2019	No	No	143	41	NEC as per 4th Edition of Practice of Neonatology	Surgery	Fibrinogen	Yes	2.496 g/L	Diagnosis	No
Fundora 2022[101]	USA	2015 - 2019	Yes	Yes	32	14	Bell's II and III	Surgery	Plasma Gal-4	Yes	>0.7 ng/ml and >1.38 ng/ml	Diagnosis	No
Palleri 2022[102]	Sweden	2009 - 2014	No	No	34	54	Bell's II and III	Surgery, severe inflammation on histology or death from NEC	Plasma sodium	Yes	<135 mmol/L	Diagnosis	No
Sharif 2020[103]	UK	2009 - 2015	No	No	64	68	Bell's II	Surgery	Serum albumin	Yes	≤20 g/L	Day 1 of diagnosis	No
Cakir 2020[34]	Turkey	2014 - 2017	No	Yes	9	8	Bell's II and III	Surgery	Serum interleukin 33	Yes	3.1 ng/ml	Diagnosis	No
Benkoe 2014[100]	Austria	2003 - 2010	No	No	63	50	Clinical concern of NEC	Surgery	Serum interleukin 8	Yes	1783 pg/ml	Diagnosis	No

Study	***Country***	***Years***	***Multicentre***	***Prospective***	***Medical (n)***	***Surgical (n)***	***Inclusion criteria***	***Reference standard***	***Index test summary***	***Repeatable***	***Threshold (points or features)***	***Timing***	***Train and test cohort***

Table shows study demographics and index test features. n = number, UK = United Kingdom, USA = United States of America, NEC = necrotising enterocolitis, ng/g = nanogram per gram, pg/nmol = petagram per nanomole, ng/mL = nanogram per millilitre, mg/l = milligram per litre, CRP = c reactive protein, NS = not specified, pg/ml = petagram per millilitre, L = litre, mm = millimetres, mmol/L = millimole per litre, g/L = grams per litre.

Supplementary table 4 - effectiveness of single biomarker methods.

					Test effectiveness					
Study	***Index test summary***	***Threshold (points or features)***	***Timing***	***Timing of reapplication***	***Sens (%)***	***Spec (%)***	***PPV (%)***	***NPV (%)***	***AUC (%)***	***Reapplied test***
Evennett 2010[31]	Urinary intestinal fatty acid–binding protein to creatinine ratio	NS	Diagnosis	No	Infants with more severe NEC had significantly higher preoperative urinary i-FABP:Cr (7.4 pg/mmol [2.1-35.0 pg/mmol]) compared with those infants with focal disease (1.1 pg/mmol [0.3-1.7 pg/mmol], P = .002)					
Thuijls 2010[8]	Urinary intestinal fatty acid–binding protein to creatinine ratio	6.38 pg/nmol	Diagnosis	No	100	86			0.96	
Schurink 2015[33]	Urinary intestinal fatty acid–binding protein to creatinine ratio	687 ng/mL	Diagnosis	8 hours	67	75				Sens = 71, Spec = 80
Heida 2015[32]	Urinary and plasma intestinal fatty acid–binding protein	NS	Diagnosis	No	Positive linear correlation (urine - Spearman Rho 0.92, plasma - 0.68)					

Study	*Index test summary*	*Threshold (points or features)*	*Timing*	*Timing of reapplication*	*Sens (%)*	*Spec (%)*	*PPV (%)*	*NPV (%)*	*AUC (%)*	*Reapplied test*
El-Abd Ahmed 2020[30]	Urinary intestinal fatty acid–binding protein to creatinine ratio	> 2.93 ng/g	Diagnosis	No	90	92	81.8	95.8	0.864	
Schurink 2015[33]	Plasma Intestinal fatty acid-binding protein	53 ng/mL	Diagnosis	8 hours	67	75				Sens = 88, Spec =80
El-Abd Ahmed 2020[30]	Plasma Intestinal fatty acid-binding protein	3.24 ng/ml	Diagnosis	No	90	72	52.6	94.7	0.768	
Tayman 2011[36]	Plasma CRP	NS	Diagnosis	Day 3 and Day 7					0.687	AUC = 0.824 (D3) and 0.844 (D7)
Wang 2018[37]	Plasma CRP	14.6 mg/l	Before treatment of NEC	Day after treatment	87.5	72.7			0.818	Sens = 75, Spec = 86.4, AUC = 0.83

Study	*Index test summary*	*Threshold (points or features)*	*Timing*	*Timing of reapplication*	*Sens (%)*	*Spec (%)*	*PPV (%)*	*NPV (%)*	*AUC (%)*	*Reapplied test*
Cakir 2020[34]	Plasma CRP	15.7 mg/l	Diagnosis	Day 3 and Day 7	66.7	33.3			0.25	Sens = 64.3, Spec = 35.7, AUC = 0.486
Yu 2022[38]	Plasma CRP	18.5 mg/L	Diagnosis	No	75	83.3			0.783	
Srinivasjois 2010[35]	Plasma CRP change	≥390%	Diagnosis to 72hrs	No	93	88			0.933	
Morecroft 1994[57]	Plasma interleukin 6	NS	Diagnosis	No	Mean IL-6 greater in Bell's III than II 3127 pg/mL v 127					
Tayman 2011[36]	Serum interleukin 6	NS	Diagnosis	Day 3 and Day 7					0.657	AUC = 0.847 (D3) and 0.861 (D7)
Wisgrill 2019[58]	Serum interleukin 6	1440 pg/ml	Diagnosis	No	91.7	83.3			0.931	

Study	*Index test summary*	*Threshold (points or features)*	*Timing*	*Timing of reapplication*	*Sens (%)*	*Spec (%)*	*PPV (%)*	*NPV (%)*	*AUC (%)*	*Reapplied test*
Cakir 2020[34]	Serum interleukin 6	107 pg/ml	Diagnosis	Day 3 and Day 7	100	73.4			0.667	Sens = 100, Spec = 83.3, AUC = 1
Ververidis 2001[60]	Consecutive platelet counts	2 tests <100 x 10^9/L	Diagnosis onwards	No	69	60	89	29		
Ververidis 2001[60]	Platelet count trajectory	>150 x 10^9/L drop in 24 hours reaching ≤ 100 x 10^9/L	Diagnosis onwards	No	32	89	92	24		
Kenton 2005[59]	Platelet count	100,000/mm^3	Diagnosis	No	Surgical NEC in 80.8% if less than threshold versus 36.6 % if below threshold					
Tayman 2011[36]	Serum amyloid A	NS	Diagnosis	Day 3 and Day 7					0.843	AUC = 0.750 (D3) and 0.826 (D7)
Coufal 2020[61]	Serum amyloid A	NS	Diagnosis	No					0.779	

Study	Index test summary	Threshold (points or features)	Timing	Timing of reapplication	Sens (%)	Spec (%)	PPV (%)	NPV (%)	AUC (%)	Reapplied test
Wang 2018[37]	Plasma Procalcitonin	1.4 ng/mL	Before treatment of NEC	Day after treatment	75	59.1			0.534	Sens = 100, Spec = 72.7, AUC = 0.78
Yu 2022[38]	Plasma Procalcitonin	1.825 ng/ml	Diagnosis	No	75	90.7			0.864	
Stone 2013[62]	Heart rate characteristic index	NA	Continuous including prior to NEC diagnosis	No	Baseline HRC index from 1 to 3 days prior to diagnosis of NEC higher in surgical group, significant rise in HRC index prior to diagnosis of NEC (16hours in surgical group and 6hours in medical), HRC index higher at diagnosis in surgical group					
van der Schyff 2018[63]	Mean heart rate	NA	Continuous	No	Mean HR greater in surgical group up to 2 days prior to surgical intervention					
Srinivasjois 2010[35]	Lactate change	≥147%	Diagnosis to 48hrs	No	82	80			0.818	
Yu 2022[38]	Lactate	1.25 mmol/L	Diagnosis	No	90	35.2			0.636	

Study	*Index test summary*	*Threshold (points or features)*	*Timing*	*Timing of reapplication*	*Sens (%)*	*Spec (%)*	*PPV (%)*	*NPV (%)*	*AUC (%)*	*Reapplied test*
Klein 1986[64]	Red cell T-Cryptantigen activation	T-activated vs not	Diagnosis	No	61.9	90.2	86.9	69.2		
Hall 2002[65]	Red cell T-Cryptantigen activation	T-activated vs not	Diagnosis	No	TCA in 30% of stage III v 4% stage II					
Tayman 2011[36]	C5a	NS	Diagnosis	Day 3 and Day 7					0.833	AUC = 0.802 (D3) and 0.806 (D7)
Cakir 2020[34]	Endocan	1114.65 ng/ml	Diagnosis	Day 3 and Day 7	100	83.3			1	Sens = 100, Spec 85.6, AUC = 0.833
Yu 2022[38]	Fibrinogen	2.496 g/L	Diagnosis	No	65	75.9			0.72	
Fundora 2022[101]	Plasma Gal-4	>0.7 ng/ml and >1.38 ng/ml	Diagnosis	No	71% and 64%	89% and 96%			0.84	
Palleri 2022[102]	Plasma sodium	<135 mmol/L	Diagnosis	No	Adjusted odds ratio for severe NEC 4.75 (1.69-13.6)					

Study	*Index test summary*	*Threshold (points or features)*	*Timing*	*Timing of reapplication*	*Sens (%)*	*Spec (%)*	*PPV (%)*	*NPV (%)*	*AUC (%)*	*Reapplied test*
Sharif 2020[103]	Serum albumin	≤20 g/L	Day 1 of diagnosis	Day 2	25	94.92	82.35	57.14	0.587	Sens = 40.98, Spec = 83.3, PPV = 71.43, NPV = 58.14, AUC =0.656
Cakir 2020[34]	Serum interleukin 33	3.1 ng/ml	Diagnosis	Day 3 and Day 7	100	55.6			0.838	Sens = 100, Spec = 94.4, AUC = 0.991
Benkoe 2014[100]	Serum interleukin 8	1783 pg/ml	Diagnosis	No	90.5	59.2			0.82	

Table shows effectiveness of methods using a single biomarker at identifying surgical necrotising enterocolitis (NEC). Sens = sensitivity, spec = specificity, PPV = positive predictive value, NPV = negative predictive value, AUC = area under receiver operating characteristic curve, NEC = necrotising enterocolitis, i-FABP = intestinal fatty acid–binding protein, Cr = creatinine, NS = not specified, IL6 = interleukin 6, HRC = heart rate characteristic, HR = heart rate, TCA = T Cryptantigen Activation, ng/g = nanogram per gram, pg/nmol = petagram per nanomole, ng/mL = nanogram per millilitre, mg/l = milligram per litre, CRP = c reactive protein, NS = not specified, pg/ml = petagram per millilitre, L = litre, mm = millimetres, mmol/L = millimole per litre, g/L = grams per litre.

Study	***Index test summary***	***Threshold (points or features)***	***Timing***	***Timing of reapplication***	***Sens (%)***	***Spec (%)***	***PPV (%)***	***NPV (%)***	***AUC (%)***	***Reapplied test***

Supplementary table 5 - study and test characteristics of methods reporting an imaging method.

Study	***Country***	***Years***	***Multicentre***	***Prospective***	***Medical (n)***	***Surgical (n)***	***Inclusion criteria***	***Reference standard***	***Index test summary***	***Repeatable***	***Threshold (points or features)***	***Timing***	***Train and test cohort***
Palleri 2017[19]	Sweden	2010 - 2013	No	No	14	11	Bell's II and III	Surgery	US	Yes		Diagnosis	No
Bömelburg 1992[14]	Germany	1987 - 1990	No	No	16	11	Clinical concern of NEC	Surgery included delayed surgery for stricture	US	Yes		Diagnosis	No
Silva 2007[20]	Canada	2003 - 2005	No	No	18	22	Clinical and radiological features of NEC	Surgery included delayed surgery for stricture	US	Yes	3	Either diagnosis or to aid decision making	No
Chen 2018[15]	China	2010 - 2016	No	No	47	39	Bell's II and III	Surgery or death	US	Yes		Diagnosis	No
Yang 2016[22]	China	2013 - 2015	No	No	58	26	Bell's I, II and III	Surgery or death	US	Yes		Diagnosis	No
Garbi-Goutel 2014[16]	France	2009 - 2011	No	No	69	26	Clinical and radiological features of NEC	Surgery or death	US	Yes	2	Diagnosis	No

Study	***Country***	***Years***	***Multicentre***	***Prospective***	***Medical (n)***	***Surgical (n)***	***Inclusion criteria***	***Reference standard***	***Index test summary***	***Repeatable***	***Threshold (points or features)***	***Timing***	***Train and test cohort***
Wang 2016[21]	China	2014 - 2015	No	No	103	55	Bell's I, II and III	Surgery or death	US	Yes		Diagnosis	No
Muchantef 2013[18]	USA	2007 - 2011	No	No	22	33	Clinical concern of NEC	Surgery or death	US	Yes		Diagnosis	No
Yikilmaz 2014[23]	Canada	2013 - 2014	No	Yes	20	6	Bell's II and III	Surgery with necrosis	US	Yes		Diagnosis	No
Lindley 1986[17]	USA	1984 - 1985	No	No	12	3	Clinical and radiological features of NEC	Surgery with necrosis	US	Yes		Diagnosis	No
Aliev 2017[13]	Uzbekistan	NS	No	Yes	35	16	Bell's I, II and III	Surgery with necrosis requiring resection	US	Yes		Diagnosis	No
Dilli 2011[66]	Turkey	2007 - 2009	No	Yes	28	12	Bell's II and III	Surgery	US and AXR	Yes		Diagnosis	No
He 2016[67]	China	2000 - 2014	No	No	184	54	Clinical and radiological features of NEC	Surgery	US and AXR	Yes	2	Diagnosis	No

Study	Country	Years	Multicentre	Prospective	Medical (n)	Surgical (n)	Inclusion criteria	Reference standard	Index test summary	Repeatable	Threshold (points or features)	Timing	Train and test cohort
Prithviraj 2015[68]	India	2010 - 2014	No	Yes	38	22	Clinical and radiological features of NEC	Surgery included delayed surgery for stricture or death	US and AXR	Yes		Diagnosis	No
Zvizdic 2020[71]	Bosnia and Herzegovina	2008 - 2012	No	No	29	21	Bell's II and III	Bell's III	AXR	Yes		Diagnosis	No
Leonard 1982[69]	USA	1972 - 1978	No	No	13	8	Clinical concern of NEC	Surgery with necrosis or death	AXR	Yes		Diagnosis	No
Muller 2014[70]	Netherlands	2000 - 2011	No	No	86	55	Bell's II and III	Surgery (perforation or failure to respond to maximal conservative therapy) or death	AXR	Yes		Diagnosis	No
Chen 2018[15]	China	2010 - 2016	No	No	47	39	Bell's II and III	Surgery or death	AXR	Yes		Diagnosis	No

Study	*Country*	*Years*	*Multicentre*	*Prospective*	*Medical (n)*	*Surgical (n)*	*Inclusion criteria*	*Reference standard*	*Index test summary*	*Repeatable*	*Threshold (points or features)*	*Timing*	*Train and test cohort*
Coursey 2009[72]	USA	2000 - 2007	No	No	86	43	Bell's I, II and III	Surgery	DAAS AXR score	Yes		Diagnosis	No
Lin 2012[73]	China	2005 - 2011	No	No	46	15	Bell's I, II and III	Surgery	DAAS AXR score	Yes		Diagnosis	No
Yu 2022[28]	China	2015 - 2019	No	No	143	41	Bell's II and III	Surgery	DAAS AXR score	Yes	≥7	Diagnosis	Test
Haase 1981[74]	USA	NS	No	No	16	7	Clinical concern of NEC	Surgery with necrosis	Radionucleotid e scan	Yes		Diagnosis	No
Çağlar 2021[75]	Turkey	2015 - 2018	No	Yes	20	14	Bell's I, II and III	Surgery with ischaemia	CT	Yes		NS	No
Maalouf 2000[76]	UK	NS	No	Yes	2	6	Clinical concern of NEC	Surgical findings	MRI	Yes		Suspicion of need for surgery	No

Table shows study demographics and index test features. n = number, USA = United States of America, NEC = necrotising enterocolitis, DAAS = duke abdominal assessment scale, US = ultrasound, AXR = abdominal radiograph, CT = computer tomography, MRI = magnetic resonance imaging.

Supplementary table 6 - effectiveness of imaging methods.

					Test effectiveness					
Study	***Index test summary***	***Threshold (points or features)***	***Timing***	***Timing of reapplication***	***Sens (%)***	***Spec (%)***	***PPV (%)***	***NPV (%)***	***AUC (%)***	***Reapplied test***
Palleri 2017[19]	US		Diagnosis		Complex fluid collection associated with surgical intervention. All other signs non significant.					
Bömelburg 1992[14]	US		Diagnosis		PVG (5/10), pneumatosis (4/8) and no pathological bowel gas surgery (2/9)					
Silva 2007[20]	US	3	Either diagnosis or to aid decision making		82	78			0.917	
Chen 2018[15]	US		Diagnosis						0.857	
Yang 2016[22]	US		Diagnosis		Surgery or death was statistically associated with pneumoperitoneum, bowel dilatation, intestinal wall thickening and ascites.					
Garbi-Goutel 2014[16]	US	2	Diagnosis		57.7	71				

Study	Index test summary	Threshold (points or features)	Timing	Timing of reapplication	Sens (%)	Spec (%)	PPV (%)	NPV (%)	AUC (%)	Reapplied test
**Wang 2016[21]**	US		Diagnosis	Every 6-12 hours	Surgery or death was statistically associated with dilatation of the intestine, bowel wall thickening and ascites.					
**Muchantef 2013[18]**	US		Diagnosis						0.88	
**Yikilmaz 2014[23]**	US		Diagnosis		100	95.4	80	100		
**Lindley 1986[17]**	US		Diagnosis		66.7	75	40	90		
**Aliev 2017[13]**	US		Diagnosis	Twice daily on day 2 and 3	Bowel wall thinning (<1.1mm), PVG, focal absence of peristalsis, absent bowel wall perfusion all associated with Bell's IIb and III. Bowel wall thickening (>2.5mm), increased perfusion early disease (I to IIa)					
**Dilli 2011[66]**	US and AXR		Diagnosis		AXR - free air on 12 patients, US - echoic free fluid (n=8), focal fluid collection (n=3).					
**He 2016[67]**	US and AXR	2	Diagnosis		75.9	68.5			0.78	

Study	Index test summary	Threshold (points or features)	Timing	Timing of reapplication	Sens (%)	Spec (%)	PPV (%)	NPV (%)	AUC (%)	Reapplied test
***Prithviraj 2015*[68]**	US and AXR		Diagnosis		Surgical NEC associated with many features - thickened intestinal wall, bowel wall thinning, pneumatosis, PVG, fixed loop, pneumoperitoneum, absent peristalsis, free fluid, fluid collection, increased or decreased bowel wall flow, increased superior mesenteric artery flow					
***Zvizdic 2020*[71]**	AXR		Diagnosis		Mean ratio greater for (a) and (b) in surgical group versus medical group					
***Leonard 1982*[69]**	AXR		Diagnosis		4/7 with fixed loop had necrotic bowel at surgery/autopsy, 4/14 without fixed loop had necrotic bowel at surgery/autopsy					
***Muller 2014*[70]**	AXR		Diagnosis		82	37	55	66		
***Chen 2018*[15]**	AXR		Diagnosis						0.745	
***Coursey 2009*[72]**	DAAS AXR score		Diagnosis		Odds ratio 1.69 for every point on scale increase				0.83	
***Lin 2012*[73]**	DAAS AXR score		Diagnosis		Those with Bell's III had score of 8.9 versus 5.3 in II and 3.2 in I					

Study	*Index test summary*	*Threshold (points or features)*	*Timing*	*Timing of reapplication*	*Sens (%)*	*Spec (%)*	*PPV (%)*	*NPV (%)*	*AUC (%)*	*Reapplied test*
Yu 2022[28]	DAAS AXR score	≥7	Diagnosis	No	48.7	95	73.6	86.6		
Haase 1981[74]	Radionucleotide scan		Diagnosis		2 false positive and 2 false negative. Overall error rate 12%					
Çağlar 2021[75]	CT		NS		100	100	100	100		
Maalouf 2000[76]	MRI		Suspicion of need for surgery		1 had segmental volvulus and not NEC, 4/5 with NEC required resection as bowel non viable. Fluid level in bowel, intramural gas and bubble-like appearance of intestinal wall in all that required resection and not seen in those not requiring resection.					

Table shows effectiveness of methods using an imaging method at identifying surgical necrotising enterocolitis (NEC). Sens = sensitivity, spec = specificity, PPV = positive predictive value, NPV = negative predictive value, AUC = area under receiver operating characteristic curve, NEC = necrotising enterocolitis, DAAS = duke abdominal assessment scale, US = ultrasound, AXR = abdominal radiograph, CT = computer tomography, MRI = magnetic resonance imaging, PVG = portal venous gas.

Supplementary table 7 - study and test characteristics of invasive methods.

Study	*Country*	*Years*	*Multicentre*	*Prospective*	*Medical (n)*	*Surgical (n)*	*Inclusion criteria*	*Reference standard*	*Index test summary*	*Repeatable*	*Threshold (points or features)*	*Timing*	*Train and test cohort*
Tan 2007[41]	Australia	NS	No	Yes	1$	3	Abdominal distension not responding to medical therapy	Laparotomy	Laparoscopy	Yes		Failed medical therapy	No
Leva 2010[39]	Italy	2007 - 2008	No	Yes	0	8	Bell's I and II	Laparotomy or peritoneal drain	Laparoscopy	Yes		Deterioration at 24 hours post diagnosis	No
Bellostas 2011[42]	Spain	NS	No	Yes	1$	6	Clinical concern of NEC	Laparotomy	Laparoscopy	Yes		Failed medical therapy	No
Pierro 2004[7]	UK	NS	No	Yes	2$	9	Clinical NEC not responding to medical therapy	Laparotomy	Laparoscopy	Yes		Failed medical therapy	No
Numanoglu 2011[40]	South Africa	NS	No	Yes	3$	10	Clinical concern NEC without radiological features or confirmed NEC not improving despite maximum medical therapy	Laparotomy	Laparoscopy +/- fluorescein assessment	Yes		Failed medical therapy	No

Study	*Country*	*Years*	*Multicentre*	*Prospective*	*Medical (n)*	*Surgical (n)*	*Inclusion criteria*	*Reference standard*	*Index test summary*	*Repeatable*	*Threshold (points or features)*	*Timing*	*Train and test cohort*
Pohlandt 1990[77]	Germany	NS	No	No	19	16	Bell's I and II	Surgery with gangrene	Paracentesis	Yes		NS	No
Ricketts 1986[79]	USA	1980 - 1984	No	No	2	37	Bell's II and III	Surgery with gangrene	Paracentesis	Yes		Fixed mass, fixed loop on AXR, abdominal wall erythema, clinical deterioration	No
Kosloske 1980[78]	USA	1976 - 1979	No	No	9	14	Bell's II and III	Surgery with gangrene	Paracentesis	Yes		At time of suspected gangrene	No
Tanriverdi 2013[80]	Turkey	2010 - 2012	No	Yes	9	14	Clinical and radiological features of NEC	Surgery	Intravesical pressure	Yes	6.25mmHg	Anytime post NEC diagnosis	No

Table shows study demographics and index test features. NS = not specified, n = number, $ = Rather than medical NEC indicates those that underwent laparoscopy without proceeding to laparotomy, USA = United States of America, UK = United Kingdom, mmHg = millimetres of mercury.

Supplementary table 8 - effectiveness of invasive methods.

					Test effectiveness					
Study	***Index test summary***	***Threshold (points or features)***	***Timing***	***Timing of reapplication***	***Sens (%)***	***Spec (%)***	***PPV (%)***	***NPV (%)***	***AUC (%)***	***Reapplied test***
Tan 2007[41]	Laparoscopy		Failed medical therapy	No	100	100	100	100		
Leva 2010[39]	Laparoscopy		Deterioration at 24 hours post diagnosis	No	Perforation found in 6/8 so converted to laparotomy, no perforation in 2/8 so drain inserted					
Bellostas 2011[42]	Laparoscopy		Failed medical therapy	No	No adverse outcomes, laparotomy avoided in 1/7					
Pierro 2004[7]	Laparoscopy		Failed medical therapy	No	100	100	100	100		
Numanoglu 2011[40]	Laparoscopy +/- fluorescein assessment		Failed medical therapy	No	100	100	100	100		
Pohlandt 1990[77]	Paracentesis		NS	No	100	100	100	100		

Study	Index test summary	Threshold (points or features)	Timing	Timing of reapplication	Sens (%)	Spec (%)	PPV (%)	NPV (%)	AUC (%)	Reapplied test
Ricketts 1986[79]	Paracentesis		Fixed mass, fixed loop on AXR, abdominal wall erythema, clinical deterioration	No	94.4	100	100	49.2		
Kosloske 1980[78]	Paracentesis		At time of suspected gangrene	No	91.7	100	100	88.5		
Tanriverdi 2013[80]	Intravesical pressure	6.25mmHg	Anytime post NEC diagnosis	No	100	75				

Table shows effectiveness of methods using an invasive method at identifying surgical necrotising enterocolitis (NEC). Sens = sensitivity, spec = specificity, PPV = positive predictive value, NPV = negative predictive value, AUC = area under receiver operating characteristic curve, NEC = necrotising enterocolitis, mmHg = millimetres of mercury.
